# Emergency Department Survey of Vaccination Knowledge, Vaccination Coverage, and Willingness to Receive Vaccines in an Emergency Department Among Underserved Populations — Eight U.S. Cities, April–December, 2024

**DOI:** 10.15585/mmwr.mm7429a1

**Published:** 2025-08-07

**Authors:** Robert M. Rodriguez, Jesus R. Torres, Brian Chinnock, Efrat Kean, Kristin L. Rising, Christopher Conn, Michael Gottlieb, Shwetha Sekar, Perla Gomez, Lorenia Olivera, Stephanie A. Eucker, Sofia DiFulvio, Christopher Alvarez, Melanie F. Molina, Shaokui Ge, Vijaya Arun Kumar

**Affiliations:** ^1^Department of Medicine, University of California Riverside School of Medicine, Riverside, California; ^2^Department of Emergency Medicine, David Geffen School of Medicine at UCLA, Olive View-UCLA Medical Center, Los Angeles, California; ^3^Department of Emergency Medicine, University of California San Francisco, Fresno Medical Education Program, Fresno, California; ^4^Department of Emergency Medicine, Sidney Kimmel Medical College, Thomas Jefferson University, Philadelphia, Pennsylvania; ^5^Department of Emergency Medicine, Wayne State University, Detroit, Michigan; ^6^Department of Emergency Medicine, Rush University Medical Center, Chicago, Illinois; ^7^Department of Emergency Medicine, University of California San Francisco School of Medicine, San Franscisco, California; ^8^Department of Emergency Medicine, Duke University, Durham, North Carolina.

**Summary**

**What is already known about this topic?**

U.S. adult vaccination coverage data are limited, especially among populations lacking primary health care access.

**What is added by this report?**

In a multicenter emergency department (ED) survey of vaccine knowledge, self-reported vaccination status, and willingness to receive vaccines if offered in an ED, 49.4% of non–critically ill adult participants had not heard of at least one CDC-recommended vaccine, and 85.9% had missed one or more. Overall, 46.4% of participants who were not up to date with recommended vaccines said they would accept one or more missing vaccines if offered during their ED visit; 86.7% of those participants said they would accept all missing vaccines.

**What are the implications for public health practice?**

EDs could be explored as settings to offer vaccination screening, recommendations, counseling, and referrals to increase vaccination coverage among underserved populations.

## Abstract

Current models of vaccination coverage screening and surveillance might miss underserved populations whose only health care access occurs in emergency departments (EDs). During April–December 2024, a survey of non–critically ill adult patients evaluated in 10 EDs in eight U.S. cities across five states was conducted to ascertain patients’ vaccination knowledge, self-reported vaccination coverage, and willingness to receive vaccines in an ED. Among 4,326 patients approached by the research team, 3,285 (75.9%) agreed to participate. Non-Hispanic Black or African American (Black), non-Hispanic White, and Hispanic or Latino (Hispanic) persons each accounted for approximately 30% of participants; 17.9% spoke Spanish as their primary language; 7.8% had unstable or marginal housing; and 21.0% lacked a source of primary health care. Approximately one half (49.4%) had not heard of one or more CDC-recommended vaccines for their age group, and 85.9% had not received one or more of the recommended vaccines. Factors associated with not being up to date with recommended vaccinations included non-Hispanic Black race and ethnicity (adjusted odds ratio [aOR] = 1.93; 95% CI = 1.32–2.85), lack of primary health care (aOR = 2.91; 95% CI = 1.74–5.13), and lack of health insurance (aOR = 3.01; 95% CI = 1.27–8.82). Among 2,821 participants who were not up to date with recommended vaccines, 46.4% said that they would accept one or more missing vaccines if they could be provided during their ED visit, and 86.7% of these persons said they would accept all missing vaccines. The primary reasons for missed vaccine doses were that the participant was unaware of or had not been offered the vaccines. EDs could be explored as additional sites to offer vaccination screening, recommendations, counseling, and referrals to increase vaccination coverage among underserved populations.

## Introduction

Review of patient vaccination status, coupled with counseling regarding needed vaccines, is a fundamental component of primary health care. During the previous 2 decades, the list of CDC-recommended vaccinations has grown, with recommendations for varicella zoster (shingles), respiratory syncytial virus (RSV), and COVID-19 vaccines varying by age group and risk factors adding to vaccination schedule complexity (*1*).

Approximately one third of the U.S. adult population receives no primary health care; these persons rarely, if ever, receive a comprehensive review of their vaccination status, vaccination counseling, and opportunities to receive recommended vaccines (*2*,*3*). Emergency departments (EDs), the only health care access point for millions of persons in the United States, might be positioned to address gaps in public health interventions, including vaccination messaging and delivery (*4*). In two ED-based cluster randomized controlled trials, provision of COVID-19 and influenza vaccination messaging platforms resulted in twofold to threefold increases in vaccine acceptance and receipt rates in underserved populations (*5*,*6*). Expanding this ED-based vaccination surveillance concept to examine all CDC-recommended adult vaccines, this study sought to ascertain among non–critically ill adult ED patients, 1) knowledge and receipt of CDC-recommended vaccines for their age group; 2) reasons for previously not receiving and currently not accepting recommended vaccines; and 3) willingness to accept missing vaccines if they were offered in an ED.

## Methods

### Pilot Testing and Institutional Review Board Review

Initial survey templates were reviewed with a patient community advisory board, and the survey instrument was pilot tested with six ED patients to assess comprehension and response consistency. This research was reviewed and approved by a central institutional review board (IRB) with reliance at five of the ED sites and by individual IRBs at the other five ED sites.*

### Study Sites and Sample Size

This study was conducted during April 18–December 31, 2024, at 10 EDs in eight U.S. cities in five states (Chicago, Illinois; Detroit, Michigan; Durham, North Carolina; Fresno, California; Los Angeles, California; Philadelphia, Pennsylvania; San Francisco, California; and Sylmar, California). Together, these EDs reported an estimated 637,392 ED visits in 2024. A priori sample size calculations determined that 2,401 patients (across all sites) needed to be enrolled for the lower and upper bounds of the 95% CIs around the point estimates of the primary outcomes (knowledge and receipt of age-recommended vaccines) to be within a 2% margin of error. To balance enrollment across sites, the study stopped enrollment at any one of the 10 EDs if that ED reached 400–450 enrollees. Strengthening the Reporting of Observational studies in Epidemiology (STROBE) guidelines were followed (*7*).

### Patient Enrollment and Survey Administration

During weekdays (typically 9:00 a.m.–5:00 p.m., Monday–Friday), research staff members reviewed ED patient care dashboards (lists of patients in the ED with their age and primary symptoms) to identify adults aged ≥18 years who met inclusion and exclusion criteria^†^; they then offered enrollment to eligible patients using a scripted consent form. Age-specific surveys (i.e., for persons aged 18–26,^§^ 27–49, 50–64, and ≥65 years) were administered in English, Spanish, or (at two sites) Mandarin (Supplementary Box). Participants were asked to respond to the following questions about each CDC-recommended vaccine for their age group as of April 2024 (*7*): 1) “Have you heard about [VACCINE]?”; 2) “Have you received [VACCINE]?”; 3) (If no), “Why have you not received [VACCINE]?”; and 4) “Would you accept any of these [MISSING VACCINES] today during your ED visit if they could be provided to you?” Plain language accompanied by explanations was used whenever possible to describe the recommended vaccines (e.g., “shingles” rather than “varicella zoster”). Age thresholds for survey questions and time thresholds for up-to-date status were determined using the CDC Recommended Adult Immunization Schedule (as of April 2024)^¶^ (*1*). For example, age ≥60 years was used to prompt the series of questions about RSV,** pneumococcal, shingles, COVID-19, influenza, and tetanus-containing vaccines; tetanus vaccination was considered up to date only if the most recent dose had been received within the previous 10 years. In addition to vaccination questions, participants were asked questions about their race, ethnicity, health insurance, primary care, and housing.^††^

### Analysis

Participant characteristics were summarized as raw counts and frequency percentages, and aggregate key survey question responses were summarized as percentages with 95% CIs, excluding nonresponses in proportion denominators. Three multiple logistic regression models were fitted, one each with incomplete vaccination knowledge, vaccine coverage, and willingness to accept one or more missed vaccines as dependent variables; model adjustment factors included age group, sex, city, and characteristics previously associated with low vaccination coverage (Black or African American [Black] race, Hispanic or Latino [Hispanic] ethnicity, non-English primary language, no source of primary health care, and lack of health insurance) (*4*). Model results are reported as adjusted odds ratios (aORs) with 95% CIs, and two-tailed p-values; p-values <0.05 and aORs with 95% CIs that excluded 1 were considered statistically significant. Analyses were conducted using RStudio (version 2023.12.1; RStudio, Inc.).

## Results

### Participant Characteristics

Among 4,326 non–critically ill patients approached in the 10 participating EDs, 3,285 (75.9%) agreed to participate. Among these, 54.6% were female; 29.6% were Black, 27.0% were non-Hispanic White, and 31.5% were Hispanic; 7.8% had marginal or unstable housing; 21.0% lacked primary health care; and for 17.9%, Spanish was the primary language (Table 1).

**TABLE 1:** Characteristics of participants* aged ≥18 years in vaccination survey — 10 emergency departments in eight U.S. cities,^†^ April 18–December 31, 2024

Characteristic	Age group, yrs, no. (%)				
	All N = 3,285	18–26 n = 422	27–49 n = 1,235	50–64 n = 802	≥65 n = 826
**Median age, yrs (IQR)**	**49.4 (33–65)**	**22.7 (21–25)**	**37.5 (32–43)**	**57.3 (54–61)**	**72.3 (68–78)**
**Sex**					
Female	**1,792 (54.6)**	268 (63.5)	710 (57.5)	365 (45.5)	449 (54.4)
Male	**1,459 (44.4)**	145 (34.4)	511 (41.5)	430 (53.6)	372 (45.0)
Other/Prefer not to answer	**34 (1.0)**	9 (2.1)	12 (10.1)	7 (0.9)	5 (0.6)
**Housing status^§^**					
Stable	**3,022 (92.2)**	404 (95.7)	1,114 (90.4)	717 (89.6)	787 (95.7)
Marginal	**102 (3.1)**	10 (2.4)	47 (3.8)	31 (3.9)	14 (1.7)
Unstable	**153 (4.7)**	8 (1.9)	72 (5.8)	52 (6.5)	21 (2.6)
**Race and ethnicity^¶^**					
Asian	**125 (3.8)**	34 (8.2)	40 (3.2)	19 (2.4)	32 (3.9)
Black or African American	**967 (29.6)**	110 (26.4)	308 (25.0)	277 (34.6)	272 (33.1)
Native American	**19 (0.6)**	2 (0.5)	7 (0.6)	5 (0.6)	5 (0.6)
Native Hawaiian or Pacific Islander	**19 (0.6)**	2 (0.5)	11 (0.9)	4 (0.5)	2 (0.2)
White	**882 (27.0)**	94 (22.5)	277 (22.5)	195 (24.3)	316 (38.5)
Hispanic or Latino	**1,030 (31.5)**	142 (34.1)	487 (39.5)	247 (30.8)	154 (18.8)
Other/Mixed	**230 (7.0)**	33 (7.9)	103 (8.4)	54 (6.7)	40 (4.9)
**Heath insurance**					
Affordable Care Act	**51 (1.6)**	12 (2.8)	39 (3.2)	0 (—)	0 (—)
Community health plan (e.g., Healthy San Francisco)	**43 (1.4)**	2 (0.5)	20 (1.6)	15 (2.2)	6 (0.8)
Medicaid/MediCal	**956 (30.5)**	140 (33.2)	466 (37.7)	246 (35.4)	104 (13.2)
Medicare	**584 (18.6)**	18 (4.3)	69 (5.6)	117 (16.9)	380 (48.3)
Military/Veterans Administration	**6 (0.2)**	1 (0.2)	4 (0.3)	0 (—)	1 (0.1)
No health insurance	**251 (8.0)**	51 (12.1)	179 (14.5)	7 (1.0)	14 (1.8)
Private	**1,077 (34.3)**	161 (38.2)	385 (31.2)	273 (39.3)	258 (32.8)
Other	**169 (5.4)**	37 (8.8)	73 (5.9)	36 (5.2)	23 (2.9)
**Primary language**					
English	**2,601 (79.2)**	368 (87.2)	950 (76.9)	613 (76.4)	670 (81.1)
Spanish	**588 (17.9)**	39 (9.2)	257 (20.8)	167 (20.8)	125 (15.1)
Other	**96 (2.9)**	15 (3.6)	28 (2.2)	22 (2.7)	31 (3.8)
**Have primary care physician or clinic**					
No	**689 (21.0)**	130 (30.8)	319 (25.9)	173 (21.7)	67 (8.1)
Yes	**2,585 (79.0)**	292 (69.2)	912 (74.1)	625 (78.3)	756 (91.9)
**City of ED visit^†^**					
Chicago	**403 (12.3)**	31 (7.3)	152 (12.3)	98 (12.2)	122 (14.8)
Detroit	**287 (8.7)**	33 (7.8)	106 (8.6)	70 (8.7)	78 (9.4)
Durham	**256 (7.8)**	33 (7.8)	71 (5.7)	56 (7.0)	96 (11.6)
Fresno	**468 (14.2)**	73 (17.3)	242 (19.6)	81 (10.1)	72 (8.7)
Los Angeles	**399 (12.1)**	73 (17.3)	118 (9.6)	93 (11.6)	115 (13.9)
Philadelphia	**402 (12.2)**	100 (23.7)	99 (8.0)	103 (12.8)	100 (12.1)
San Francisco	**673 (20.5)**	52 (12.3)	276 (22.3)	169 (21.1)	176 (21.3)
Sylmar	**397 (12.1)**	27 (6.4)	171 (13.8)	132 (16.5)	67 (8.1)

**Abbreviation**: ED = emergency department.

* Non–critically ill adults aged ≥18 years evaluated in an ED.

^†^ Chicago, Illinois; Detroit, Michigan; Durham, North Carolina; Fresno, California; Los Angeles, California; Philadelphia, Pennsylvania; San Francisco, California; and Sylmar, California.

^§^ Patients were asked, “What is your living situation today?” Stable was defined as a response of, “I have a steady place to live (home, apartment, or other).” Marginal was defined as a response of, “I have a place to live today, but I am worried about losing it in the future.” Unstable was defined as a response of, “I do not have a steady place to live (I am temporarily staying with others, in a hotel, in a shelter, living outside on the street, on a beach, in a car, abandoned building, bus or train station, or in a park).”

**^¶^** Persons of Hispanic or Latino (Hispanic) origin might be of any race but are categorized as Hispanic; all racial groups are non-Hispanic.

### Participant Knowledge About Vaccines

Approximately one half (49.4%; 95% CI = 47.7%–54.1%) of participants had not heard of one or more recommended vaccines (Table 2). In adjusted models, the following characteristics were associated with not having heard of one or more vaccines: male sex (aOR = 2.01; 95% CI = 1.71–2.34), Black race (aOR = 2.01; 95% CI = 1.62–2.48), Hispanic ethnicity (aOR = 1.94; 95% CI = 1.51–2.48), non-English primary language (aOR = 2.42; 95% CI = 1.82–3.22), lack of primary health care (aOR = 1.68; 95% CI = 1.35–2.08), and being uninsured (aOR = 1.56; 95% CI = 1.14–2.16) (Table 3).

**TABLE 2:** Percentage of participants aged ≥18 years who had not heard of or had missed one or more recommended vaccines and who would accept one or more missing vaccines if offered in an emergency department, by age group — 10 emergency departments in eight U.S. cities,* April 18–December 31, 2024

Age group, yrs	% of participants (95% CI)		
	Had not heard of one or more vaccines	Had not received one or more vaccines	Would accept one or more missing vaccines
**Total**	**49.4 (47.7–51.1)**	**85.9 (84.8–87.2)**	**46.4 (44.6–48.2)**
18–26	51.2 (46.5–55.9)	78.4 (74.3–82.6)	42.0 (36.7–47.3)
27–49	47.9 (45.1–50.6)	86.4 (84.5–88.3)	45.6 (42.6–48.6)
50–64	48.6 (45.2–52.1)	89.8 (87.7–91.9)	49.9 (46.3–53.6)
≥65	51.5 (48.0–54.9)	85.8 (83.4–88.2)	46.2 (42.5–49.8)

* Chicago, Illinois; Detroit, Michigan; Durham, North Carolina; Fresno, California; Los Angeles, California; Philadelphia, Pennsylvania; San Francisco, California; and Sylmar, California.

**TABLE 3:** Characteristics* associated with not having heard of or having missed one or more recommended vaccines and willingness to accept one or more missing vaccines if offered in an emergency department among participants aged ≥18 years — 10 emergency departments in eight U.S. cities,^†^ April 18–December 31, 2024

Characteristic	Had not heard of one or more vaccines				Had not received one or more vaccines				Would accept one or more missing vaccines			
	OR (95% CI)	p-value	aOR (95% CI)	p-value	OR (95% CI)	p-value	aOR (95% CI)	p-value	OR (95% CI)	p-value	aOR (95% CI)	p-value
**Age group, yrs**												
18–26	1.00 (Ref)	—	1.00 (Ref)	—	1.00 (Ref)	—	1.00 (Ref)	—	1.00 (Ref)	—	1.00 (Ref)	—
27–49	0.88 (0.70–1.09)	0.24	0.71 (0.55–0.89)	0.003	1.57 (1.07–2.30)	0.02	1.64 (1.09–2.45)	0.02	1.16 (0.90–1.49)	0.24	0.92 (0.71–1.21)	0.56
50–64	0.90 (0.71–1.14)	0.40	0.69 (0.52–0.90)	0.006	2.05 (1.32–3.20)	0.001	2.34 (1.47–3.76)	<0.001	1.38 (1.06–1.79)	0.02	1.12 (0.83–1.51)	0.46
≥65	1.01 (0.80–1.28)	0.93	1.02 (0.78–1.34)	0.87	1.12 (0.75–1.65)	0.57	1.67 (1.08–2.55)	0.02	1.18 (0.91–1.54)	0.21	1.18 (0.88–1.59)	0.27
**Sex**												
Female	1.00 (Ref)	—	1.00 (Ref)	—	1.00 (Ref)	—	1.00 (Ref)	—	1.00 (Ref)	—	1.00 (Ref)	—
Male	2.02 (1.77–2.33)	<0.001	2.01 (1.71–2.34)	<0.001	1.55 (1.18–2.05)	0.002	1.28 (0.96–1.71)	0.09	1.15 (0.99–1.34)	0.07	1.09 (0.92–1.29)	0.32
Other	0.67 (0.31–1.35)	0.27	0.81 (0.32–1.90)	0.64	1.54 (0.46–9.54)	0.56	1.22 (0.34–7.89)	0.79	0.70 (0.28–1.65)	0.43	0.61 (0.21–1.59)	0.33
**Race and ethnicity^§^**												
Black or African American	1.90 (1.58–2.29)	<0.001	2.01 (1.62–2.48)	<0.001	2.40 (1.70–3.43)	<0.001	1.93 (1.32–2.85)	<0.001	1.01 (0.70–1.05)	0.14	1.01 (0.80–1.26)	0.96
White	1.00 (Ref)	—	1.00 (Ref)	—	1.00 (Ref)	—	1.00 (Ref)	—	1.00 (Ref)	—	1.00 (Ref)	—
Hispanic or Latino	2.78 (2.31–3.36)	<0.001	1.94 (1.51–2.48)	<0.001	1.97 (1.43–2.75)	<0.001	1.45 (0.91–2.36)	0.12	1.21 (1.61–2.40)	0.001	1.21 (0.93–1.58)	0.16
Mixed	1.13 (0.80–1.60)	0.48	1.03 (0.71–1.48)	0.88	1.92 (1.03–4.01)	0.06	1.38 (0.71–2.94)	0.37	1.42 (0.98–2.05)	0.06	1.42 (0.97–2.08)	0.07
Other	1.61 (1.20–2.15)	0.001	1.21 (0.87–1.65)	0.27	1.58 (0.96–2.75)	0.08	1.24 (0.72–2.27)	0.46	1.09 (0.85–1.60)	0.36	1.09 (0.77–1.55)	0.61
**Primary language**												
English	1.00 (Ref)	—	1.00 (Ref)	—	1.00 (Ref)	—	1.00 (Ref)	—	1.00 (Ref)	—	1.00 (Ref)	—
Not English	2.44 (2.05–2.92)	<0.001	2.42 (1.82–3.22)	<0.001	1.18 (0.85–1.66)	0.34	1.42 (0.83–2.48)	0.30	2.75 (2.28–3.33)	<0.001	1.09 (0.81–1.45)	0.57
**Have primary care physician**												
Yes	1.00 (Ref)	—	1.00 (Ref)	—	1.00 (Ref)	—	1.00 (Ref)	—	1.00 (Ref)	—	1.00 (Ref)	—
No	2.45 (2.06–2.93)	<0.001	1.68 (1.35–2.08)	<0.001	3.57 (2.26–6.02)	<0.001	2.91 (1.74–5.13)	<0.001	1.29 (1.08–1.54)	0.006	0.83 (0.66–1.05)	0.12
**Have health insurance**												
Yes	1.00 (Ref)	—	1.00 (Ref)	—	1.00 (Ref)	—	1.00 (Ref)	—	1.00 (Ref)	—	1.00 (Ref)	—
No	2.42 (1.84–3.20)	<0.001	1.56 (1.14–2.16)	0.006	5.50 (2.31–17.9)	<0.001	3.01 (1.27–8.82)	0.02	1.51 (1.15–1.99)	0.003	1.15 (0.82–1.61)	0.41
**City^†^**												
Chicago	0.95 (0.74–1.21)	0.67	1.06 (0.81–1.40)	0.66	2.55 (1.35–5.25)	0.006	2.58 (1.34–5.38)	0.007	0.35 (0.27–0.46)	<0.001	0.35 (0.26–0.46)	<0.001
Detroit	0.99 (0.75–1.31)	0.95	1.04 (0.75–1.42)	0.83	2.50 (1.23–5.79)	0.02	2.24 (1.05–5.34)	0.05	0.61 (0.46–0.81)	<0.001	0.62 (0.45–0.85)	0.003
Durham	0.46 (0.34–0.62)	<0.001	0.52 (0.37–0.73)	<0.001	0.24 (0.16–0.37)	<0.001	0.28 (0.18–0.45)	<0.001	0.36 (0.25–0.51)	<0.001	0.37 (0.25–0.53)	<0.001
Fresno	0.98 (0.77–1.24)	0.83	1.11 (0.84–1.44)	0.48	1.79 (1.04–3.21)	0.04	1.95 (1.09–3.59)	0.03	0.64 (0.49–0.82)	<0.001	0.61 (0.45–0.79)	<0.001
Los Angeles	0.80 (0.62–1.02)	0.08	0.92 (0.71–1.21)	0.53	0.85 (0.53–1.38)	0.50	1.04 (0.64–1.69)	0.88	0.71 (0.54–0.93)	0.01	0.66 (0.51–0.88)	0.004
Philadelphia	0.50 (0.38–0.64)	<0.001	0.58 (0.44–0.77)	<0.001	0.71 (0.45–1.12)	0.13	0.83 (0.51–1.35)	0.45	0.48 (0.36–0.63)	<0.001	0.46 (0.34–0.63)	<0.001
San Francisco	1.00 (Ref)	—	1.00 (Ref)	—	1.00 (Ref)	—	1.00 (Ref)	—	1.00 (Ref)	—	1.00 (Ref)	—
Sylmar	1.26 (0.98–1.62)	0.07	0.43 (0.31–0.61)	<0.001	0.72 (0.46–1.14)	0.16	0.36 (0.21–0.65)	<0.001	5.19 (3.70–7.42)	<0.001	5.25 (3.44–8.22)	<0.001

**Abbreviations:** aOR = adjusted odds ratio; ED = emergency department; OR = odds ratio; Ref = referent group.

* The three multiple logistic regression models were fitted with the following outcomes: 1) not having heard of one or more vaccines, 2) not having received one or more vaccines (not being up to date with recommended vaccinations), and 3) willingness to accept one or more vaccines if offered in an ED as the dependent variables, and age groups as independent variables. Model adjustment factors included sex, city, and characteristics previously associated with low vaccination coverage (Black or African American race, Hispanic or Latino [Hispanic] ethnicity, non-English primary language, no primary health care, and no health insurance). Insurance status data were missing for 1.46% respondents, race and ethnicity data were missing for 0.40%, and having a primary care physician data were missing for 0.33%; all other covariates had no missing data.

**^†^** Chicago, Illinois; Detroit, Michigan; Durham, North Carolina; Fresno, California; Los Angeles, California; Philadelphia, Pennsylvania; San Francisco, California; and Sylmar, California.

^§^ Persons of Hispanic origin might be of any race but are categorized as Hispanic; all racial groups are non-Hispanic.

### Participant Self-Reported Vaccine Receipt

Overall, 85.9% (95% CI = 84.8%–87.2**%**) of participants reported that they had not received one or more of the recommended vaccines. Associated factors included Black race (aOR = 1.93; 95% CI = 1.32–2.85), lack of primary health care (aOR = 2.91; 95% CI = 1.74–5.13), being uninsured (aOR = 3.01; 95% CI = 1.27–8.82), age ≥27 years; and having an ED visit in Chicago (aOR = 2.58; 95% CI 1.34–5.38), Detroit (aOR = 2.24; 95% CI = 1.05–5.34), or Fresno (aOR = 1.95; 95% CI = 1.09–3.59) (Table 3).

Participants had received approximately one half (51.3%) of recommended vaccines (Supplementary Table 1). Influenza vaccine was the most commonly reported missed vaccine among persons aged 18–26 years (64.5%) and 27–49 years (65.6%). Among those aged 50–64 years, shingles vaccine was most commonly missed (63.0% of participants), and among adults aged ≥65 years, the most commonly missed vaccine was RSV vaccine (74.2%). Among vaccines recommended for all age groups, 82.6% of participants reported having received COVID-19 vaccine, 42.9% had received a tetanus-containing vaccine within the previous 10 years, and 41.6% had received an influenza vaccine (Supplementary Table 2).

### Participant Willingness to Accept Vaccines if Offered in an ED

Among the 2,821 participants who were not up to date with vaccination, 1,309 (46.4%; 95% CI = 44.6%–48.2%) said they would accept one or more missed vaccines if they were offered in an ED (Table 2); among these persons, 1,135 (86.7%; 95% CI = 84.8%–88.5%) said they would accept all missing vaccines. The only factor associated with more willingness to accept vaccines if offered in an ED was having an ED visit in Sylmar (aOR = 5.25; 95% CI = 3.44-8.22). 

The most common reasons participants gave for having missed recommended vaccines were that they had not heard of the vaccine (40.7%) and that they had heard of it but were unaware that the vaccine was recommended for them (9.6%). The most common reasons participants gave for stating that they would not accept vaccines if they were offered to them the same day were that they felt too ill in the ED at that time (27.4%) and that they needed more information about the vaccines and the diseases they prevent (19.3%).

## Discussion

Current models of vaccination status screening and surveillance, such as the National Health Interview Survey (NHIS), miss underserved populations whose only health care interactions occur in EDs. In this analysis, high rates of incomplete knowledge about vaccines and low self-reported vaccination coverage were observed across all ED patient age groups, especially among Black and Hispanic participants and participants who lacked a source of primary health care and health insurance. Full vaccination coverage also differed by site (lower in Chicago, Detroit, and Fresno), as did willingness to accept vaccines (higher in Sylmar). Many participants reported that they would accept all missing vaccines if they were offered in an ED. Based on the number of respondents who reported that they would accept all missing vaccines if offered (1,135), this analysis suggests that up-to-date coverage in this population could theoretically more than triple from its current level of 14% to as high as 48%.

Apart from COVID-19 and influenza vaccination coverage, data regarding U.S. adult vaccination coverage are limited. In this ED-based analysis, self-reported up-to-date vaccination coverage rates were lower across all age groups than the 22.8% rate among adults reported from the 2022 NHIS. However, NHIS had a lower response rate (47.7%), used substantially different methods (in-person household interviews), and did not include some vaccines that were included in this analysis (e.g., measles, mumps, and rubella; meningococcal; and hepatitis B vaccines) (*8*).

Considering the millions of visits to U.S. EDs (155 million in 2022) (*9*), these findings suggest that asking non–critically ill adults in EDs about their vaccination status, and offering missed vaccinations to those who express interest, might improve vaccination coverage. Although comprehensive provision of all recommended adult vaccines to ED patients is likely impractical, the development and implementation of ED-centered programs to offer recommendations for vaccination and provide vaccine counseling and referrals to vaccine providers are logical next steps. Experience from previous vaccine delivery research suggests that such programs would need to be automated and embedded into the ED workflow so that they do not interfere with or add to the workload of ED operations (*4**–**6*). 

### Limitations

The findings in this report are subject to at least three limitations. First, because no centralized registry for adult vaccination exists, documentation of receipt of vaccines from electronic medical records (EMRs) was not included in the study protocol. Nevertheless, the survey method used is standard for vaccination coverage surveillance, and other investigators have found high concordance between self-reports of vaccination and data reported in EMRs (*10*). Second, the survey questions related to vaccination receipt are subject to recency and recall biases; participants might have more easily remembered vaccines that they actively sought as senior adults (e.g., shingles vaccines) than those received at younger ages. Finally, reported vaccine acceptance rates might not reflect acceptance rates in other EDs with different populations, and they could have been affected by other factors, including social desirability bias.

### Implications for Public Health Practice

This ED-based survey identified substantial gaps in knowledge about and receipt of age-recommended vaccines and suboptimal self-reported coverage. Many patients reported that they would accept vaccines if they were offered to them in an ED. EDs could be explored as additional sites to offer vaccination screening, recommendations, counseling, and referrals to increase vaccine coverage among underserved populations.

## Footnotes

* The central IRB for the five ED sites in Durham, Fresno, Philadelphia, and San Francisco was the University of California San Francisco; the IRB for the Chicago ED site was Rush University; the IRB for the two Detroit ED sites was Wayne State University, and the IRB for the two Los Angeles ED sites was the University of California Los Angeles.

† Exclusion criteria for participation in this survey study were 1) inability to provide consent and participate in a survey because of intoxication, overdose, altered mental status, agitation, or critical illness (defined according to local standards at each site); 2) respiratory or contact isolation; 3) victim of major trauma; 4) incarceration; and 5) psychiatric hold.

§ The CDC Adult Immunization Schedule begins at age 19 years. Because the intent of this study was to examine adults by the usual adult definition of age ≥18 years, participants aged 18 years were included in the 19–26 years age group.

¶ Survey questions were based solely on age indications, without consideration of underlying medical conditions, including pregnancy. Vaccines for which shared clinical decision-making is advised (e.g., RSV vaccines for adults aged ≥60 years and human papillomavirus vaccine for adults aged 27–45 years) were considered to be recommended in this study.

** Before June 2024, RSV vaccine was recommended for adults aged ≥60 years; in June 2024, the vaccine was routinely recommended for adults aged ≥75 years and for those aged 60–74 years who were at increased risk for severe RSV disease because of certain conditions. To maintain consistency for the duration of the study, RSV vaccine was considered to be recommended for all adults aged ≥60 years. RSV Vaccine Guidance for Adults | RSV | CDC

†† Patients were asked, “What is your living situation today?” Stable was defined as a response of, “I have a steady place to live (home, apartment, or other).” Marginal was defined as a response of, “I have a place to live today, but I am worried about losing it in the future.” Unstable was defined as a response of, “I do not have a steady place to live (I am temporarily staying with others, in a hotel, in a shelter, living outside on the street, on a beach, in a car, abandoned building, bus or train station, or in a park).”
